# Tetrabenazine is neuroprotective in Huntington's disease mice

**DOI:** 10.1186/1750-1326-5-18

**Published:** 2010-04-26

**Authors:** Hongyu Wang, Xi Chen, Yuemei Li, Tie-Shan Tang, Ilya Bezprozvanny

**Affiliations:** 1 Department of Physiology, University of Texas Southwestern Medical Center at Dallas, Dallas, Texas 75390, USA; 2 State Key Laboratory of Biomembrane and Membrane Biotechnology, Institute of Zoology, Chinese Academy of Sciences, 1 Beichen West Road, Chaoyang District, Beijing 100101, PR China

## Abstract

**Background:**

Huntington's disease (HD) is a neurodegenerative disorder caused by a polyglutamine (polyQ) expansion in Huntingtin protein (Htt). PolyQ expansion in Httexp causes selective degeneration of striatal medium spiny neurons (MSN) in HD patients. A number of previous studies suggested that dopamine signaling plays an important role in HD pathogenesis. A specific inhibitor of vesicular monoamine transporter (VMAT2) tetrabenazine (TBZ) has been recently approved by Food and Drug Administration for treatment of HD patients in the USA. TBZ acts by reducing dopaminergic input to the striatum.

**Results:**

In previous studies we demonstrated that long-term feeding with TBZ (combined with L-Dopa) alleviated the motor deficits and reduced the striatal neuronal loss in the yeast artificial chromosome transgenic mouse model of HD (YAC128 mice). To further investigate a potential beneficial effects of TBZ for HD treatment, we here repeated TBZ evaluation in YAC128 mice starting TBZ treatment at 2 months of age ("early" TBZ group) and at 6 months of age ("late" TBZ group). In agreement with our previous studies, we found that both "early" and "late" TBZ treatments alleviated motor deficits and reduced striatal cell loss in YAC128 mice. In addition, we have been able to recapitulate and quantify depression-like symptoms in TBZ-treated mice, reminiscent of common side effects observed in HD patients taking TBZ.

**Conclusions:**

Our results further support therapeutic value of TBZ for treatment of HD but also highlight the need to develop more specific dopamine antagonists which are less prone to side-effects.

## Background

Huntington's disease (HD) is an inherited progressive neurodegenerative disorder characterized by chorea, gradual but inexorable cognitive decline, and psychiatric disturbances [1,2]. Selective and progressive neuronal loss of the striatal medium spiny neurons (MSN) is the major feature of neuropathological changes in HD [2]. The cause of HD is an expanded polyglutamine (polyQ) in the amino-terminus of Huntingtin (Htt), a 350 kDa ubiquitously expressed cytoplasmic protein of unknown function [3]. The cellular mechanisms underlying the cause of MSN neurodegeneration in HD are not very clear, although most experimental evidences indicate that polyQ expansion in Htt^exp ^leads to a "toxic gain of function" [4]. A number of toxic functions have been assigned to Htt^exp^, such as effects on gene transcription, formation of toxic aggregates, direct induction of apoptosis, disruption of key neuronal functions such as proteosomal or mitochondrial functions, ubiquitination pathways, axonal transport, endocytosis, synaptic transmission and calcium signaling [4-11].

In addition to glutamatergic stimulation from the cortex, the striatum is the predominant target of dopaminergic neurons that originate from the substantia nigra [12]. There is increasing evidence that the dopaminergic system may contribute to HD neuropathology [12]. Significant reduction of striatal D1 and D2 receptor density has been reported in HD patients [13-18] and HD mouse models [19-22]. High concentration of dopamine may exert direct toxic effects on striatal neurons [23-28]. Hyperdopaminergic transmission has been shown to accelerate the formation of Htt^exp ^aggregates and promote motor dysfunction in 92Q knock-in HD mouse model [29]. Recent evidences from our group and from other groups suggested that in HD dopaminergic and glutamatergic signaling pathways act synergistically to enhance the sensitivity of striatal neurons to mutant huntingtin toxicity via disturbed calcium homeostasis [30] and disregulated Cdk5 signaling [31]. All these studies pointed to an important role of dopaminergic pathway in HD and suggested that dopamine signaling pathway constitute a potential therapeutic target for HD treatment.

Tetrabenazine (TBZ) is a potent blocker of vesicular monoamine transporter (VMAT2). In multiple clinical trials TBZ has been shown to significantly reduce chorea symptoms in HD patients when compared with placebo group [32-35]. In our previous experiments with YAC128 mouse model of HD we demonstarted that long term administration of TBZ (in combination with L-dopa) alleviated motor deficits and reduced striatal cell loss in these mice [30]. In 2008 TBZ became the first drug officially approved by the Food and Drug administration for treatment of HD patients in the United States [35]. In clinical setting most HD patients would receive TBZ at the symptomatic stage after onset of the symptoms. To mimic clinical situation more precisely, we now repeated evaluation of TBZ in YAC128 HD mouse model and compared results obtained in the "early" treatment group (starting TBZ at 2 months, prior to onset of symptoms in YAC128 mice) and "late" treatment group (starting TBZ at 6 months, when motor symptoms start to develop in YAC128 mice). Our results demonstrated significant beneficial effects of TBZ in both "early" and "late" treatment groups of YAC128 HD mice.

## Results

### TBZ improves motor coordination performance of YAC128 HD mice

In the previous study we discovered that long-term oral delivery of TBZ and L-Dopa combination (starting at 2 months of age) alleviated the motor deficits in aging YAC128 mice [30]. In clinical setting most HD patients would receive TBZ at the symptomatic stage after onset of the symptoms. To compare the efficiency of "early" and "late" treatments, we now repeated TBZ trial *in vivo *with YAC128 mice model starting TBZ treatment early (2 months of age, "presymptomatic" mice) and late (6 months of age, "symptomatic" mice). The design of the trial is shown on Table 1. In our studies the mice from "early-TBZ" groups # 2 (YAC128) and # 5 (WT) were fed with 0.125 mg of TBZ suspended in 50 μl of PBS with 2% corn flour between 2 and 12 months of age (Table 1). The control groups #1 (YAC 128) and # 4 (WT) mice were fed with 2% cornflour in PBS between 2 and 12 months of age (Table 1). The "late-TBZ" groups # 3 (YAC128) and # 6 (WT) were fed with 2% cornflour in PBS between 2 and 6 months of age and then fed with 0.125 mg of TBZ suspended in 50 μl of PBS with 2% corn flour between 6 and 12 months of age (Table 1). The drugs were fed orally to mice three times a week. At 12 months of age drug delivery to all 6 groups of mice was discontinued. The effectiveness of oral drug delivery was determined by 2 months of "drug dosage" trial. We found that, 30 min after feeding, TBZ concentration in blood plasma was 24 ± 7 ng/ml, that is 1.5-fold higher than our previous study [30]. Most likely increase in blood TBZ levels is due to difference in TBZ formulations used in the current and previous studies.

**Table 1:** Design of TBZ trial in YAC128 mice.

Group #	Group name	Number of mice	Mouse genotype	Single dose (50 μl) (three times per week)	Drug dosage (mg/kg)
1	YAC-Ctrl	15	YAC128	50 μl PBS	50 μl PBS

2	YAC-TBZ (Early)	15	YAC128	0.125 mg of TBZ (started at 2 months of age)	5 mg TBZ

3	YAC-TBZ (Late)	15	YAC128	0.125 mg of TBZ (started at 6 months of age)	5 mg TBZ

4	WT-Ctrl	15	WT	50 μl PBS	50 μl PBS

5	WT-TBZ (Early)	15	WT	0.125 mg of TBZ (started at 2 months of age)	5 mg TBZ

6	WT-TBZ (Late)	15	WT	0.125 mg of TBZ (started at 6 months of age)	5 mg TBZ

Six groups of mice were used in our experiments. At 2 months of age, WT and YAC128 mice were divided into three groups each (15 mice in each group, 90 mice total, all females), and drug treatment was initiated. The drugs were fed orally to mice three times a week starting at 2 months of age for early groups and at 6 months of age for late groups. The group number, group name, number and genotype of mice in each group, and dose of single drug treatment (three times/week) are shown for each group. Also shown is the estimated drug dosage in mg/kg. Ctrl, Control; WT, wild type; YAC, YAC128.

**Figure 1:**
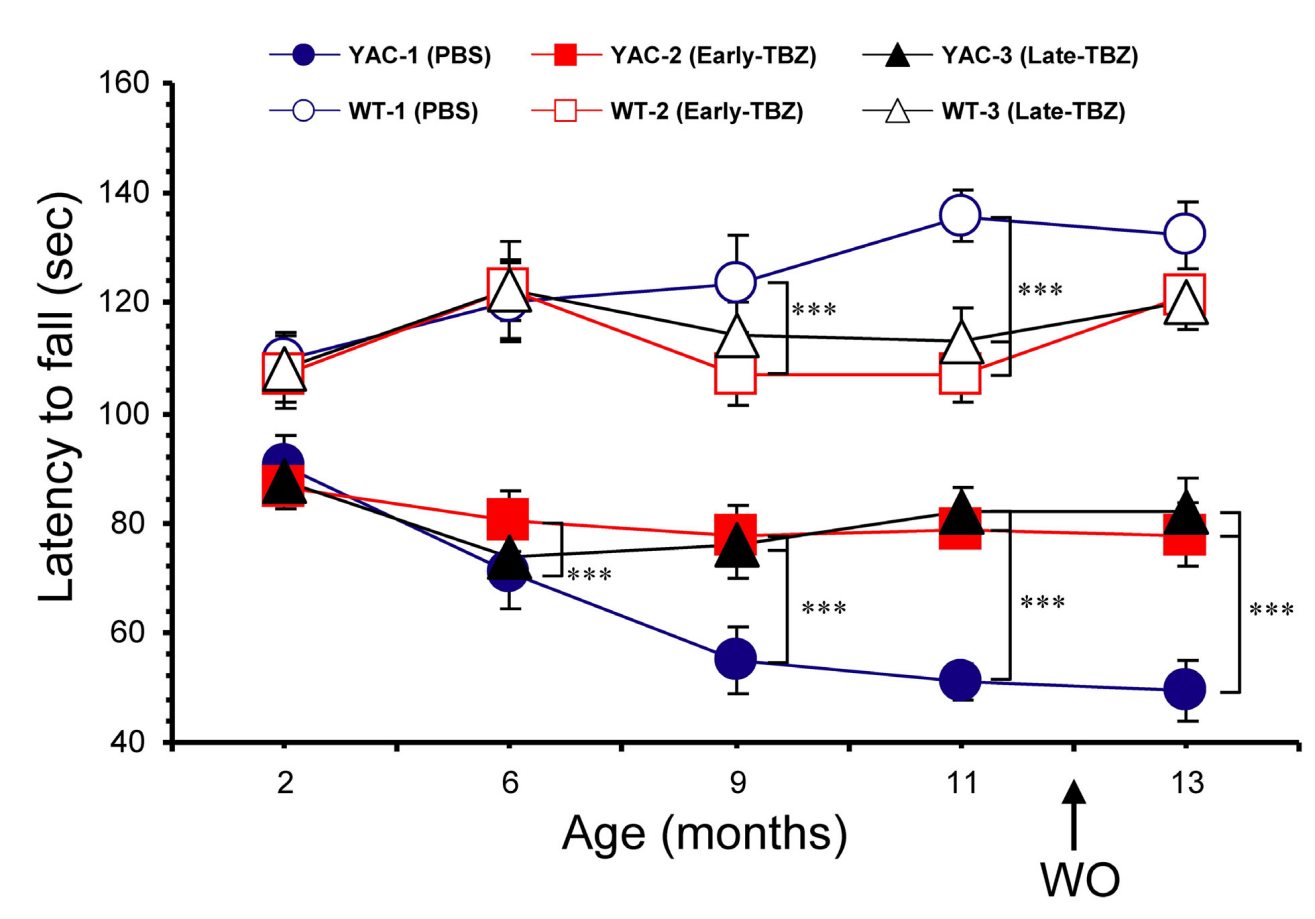
**Rotarod performance of WT and YAC128 mice**. An average latency to fall from the accelerating rotarod is shown for the WT control (Ctrl) mice (open blue circles), the YAC128 (YAC) control mice (filled blue circles), the WT mice fed with Early-TBZ (open red squares), the YAC128 mice fed with Early-TBZ (filled red squares), the WT mice fed with Late-TBZ (open black triangles), and the YAC128 mice fed with Late-TBZ (filled black triangles). For each group of mice, the results are shown as mean ± SEM at 2, 6, 9, 11 and 13 month (washout) time points. At 6, 9, 11 and 13 months of age, control WT mice performed significantly better (*p *< 0.05) than control YAC128 mice. Early-TBZ feeding to WT mice had no apparent effects on their rotarod performance at 6 months, but caused significant (*p *< 0.05) reduction at 9 and 11 months when compared to control WT group. Similar to the Early-TBZ group, Late-TBZ feeding to WT mice had no apparent effects at 9 months, but caused significant (*p *< 0.05) reduction at 11 months compared to control WT group. Early-TBZ feeding to YAC mice significantly improved (*p *< 0.05) their rotarod performance at 6, 9, 11 and 13 months when compared to control YAC128 mice. Late-TBZ feeding (6 months of age) to YAC mice significantly improved (*p *< 0.05) the rotarod performance at 9, 11 and 13 months compared to control YAC128 mice. However, both YAC-TBZ feeding groups performed significantly worse (*p *< 0.05) than any of WT groups.

The motor coordination of these 6 groups of mice was assessed by "accelerated rotarod" and "beam-walk" assays at 2, 6, 9, 11 and 13 (washout) months of age as described in our previous studies [30,36]. Basal rotarod and beam-walk performance for all groups was determined before initiation of drug feeding when the mice were 2 months of age. The length of time that the mice able to stay on accelerated rotarod was used as a measure of rotarod performance. When results were analyzed, we found that the rotarod performance of WT and YAC128 mice was similar when these mice were 2 months of age (Fig. 1). Consistent with the previous findings [30,37], control WT mice (fed with PBS) performed significantly better in rotarod assay (*p *< 0.05) than control YAC128 mice at 6, 9, 11 and 13 months of age (Fig 1). Feeding TBZ "early" to WT mice had no apparent effects on their rotarod performance at 6 months, but caused significant (*p *< 0.05) reduction at 9 and 11 months when compared to control group. Feeding TBZ "late" to WT mice had no apparent effects at 9 months, but caused significant (*p *< 0.05) reduction at 11 months when compared to control group (Fig 1). Consistent with our previous findings [30], feeding TBZ "early" to YAC128 mice significantly improved (*p *< 0.05) their rotarod performance at 6, 9, 11 and 13 months when compared to control group of YAC128 mice (Fig 1). Feeding TBZ "late" to YAC128 mice also significantly improved (*p *< 0.05) rotarod performance of these mice at 9, 11 and 13 months of age when compared to control group of YAC128 mice. However, both YAC128 TBZ feeding groups performed significantly worse (*p *< 0.05) than any of the WT groups (Fig 1).

**Figure 2:**
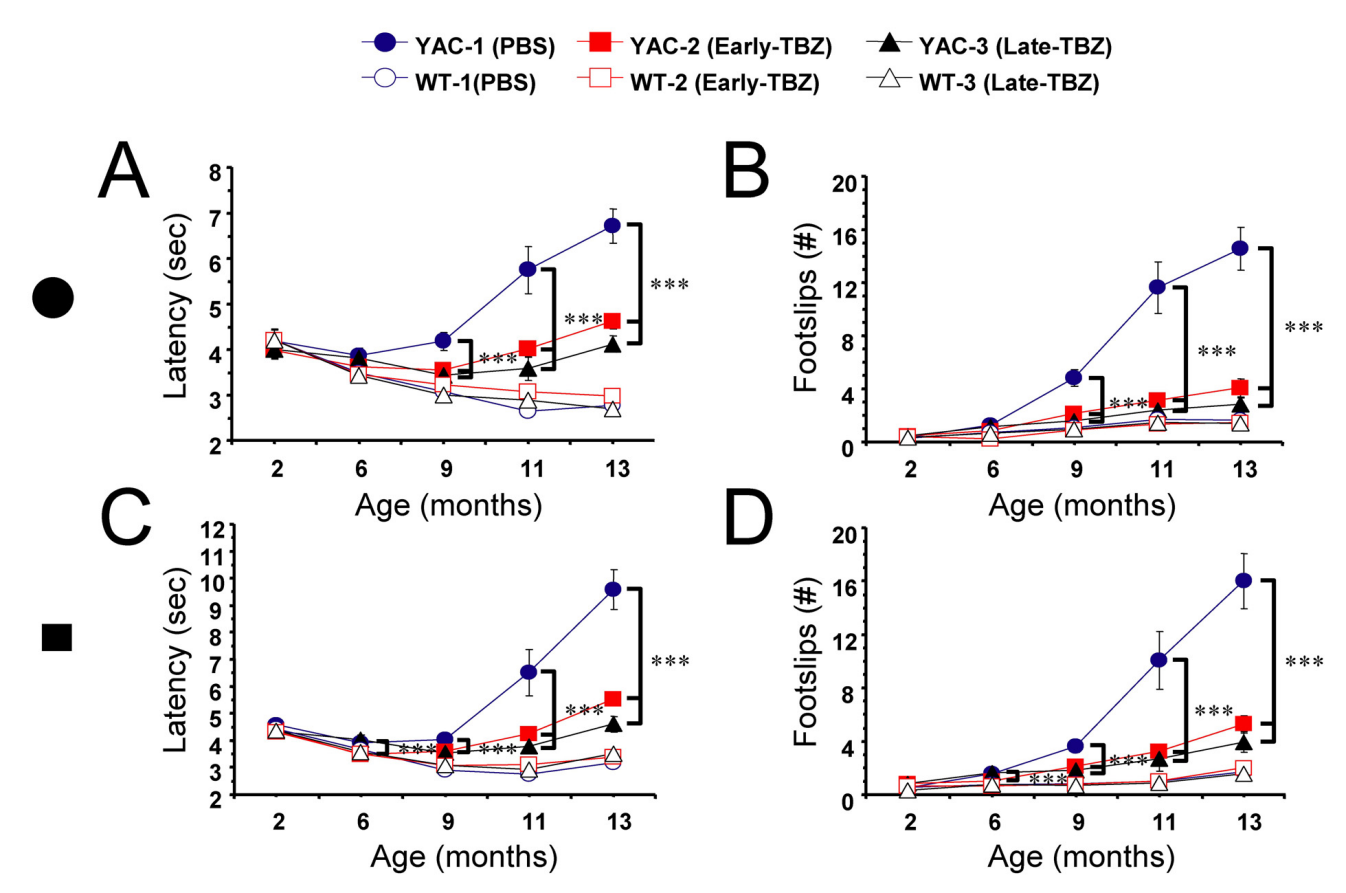
**Balance beam balance performance of WT and YAC128 mice**. The average time to cross the beam (***A***, ***C***) and the average number of foot slips on the beam (***B***, ***D***) are shown for beam-walking performed with 11 mm round beam (***A***, ***B***), and 5 mm square beam (***C***, ***D***). The data for WT control (Ctrl) mice (open blue circles), YAC128 (YAC) control mice (filled blue circles), WT mice fed with Early-TBZ (open red squares), YAC128 mice fed with Early-TBZ (filled red squares), WT mice fed with Late-TBZ (open black triangles), and YAC128 mice fed with Late-TBZ (filled black triangles) are shown as mean ± SEM at 2, 6, 8, 11 and 13 month time points. Control YAC128 mice exhibited a progressive impairment in beam-walking ability with age and beam difficulty compared with control WT mice. The significant differences (*p *< 0.05) between control YAC128 and control WT groups were observed at 9, 11 and 13 months of age on 11 mm beam; and at 6, 9, 11 and 13 months of age on 5 mm beam. Feeding TBZ to WT mice had no significant effects on beam performance compared to control WT group. Early-TBZ feeding significantly (*p *< 0.05) improved the beam performance of YAC128 mice at 9, 11 and 13 months of age on 11 mm beam, and at 6, 9,11 and 13 months of age on 5 mm beam. Late-TBZ feeding also significantly (*p *< 0.05) improved the beam performance of YAC128 mice at 9, 11 and 13 months of age on both 11 mm and 5 mm beam.

Two kinds of beams (11 mm round, and 5 mm square) were used for testing in beam-walking assay. The "latency" and "number of foot slips" were measured for each beam. When results were analyzed, we found that consistent with our previous findings [30], the control group of YAC128 mice (fed with PBS) exhibited a progressive impairment in beam-walking ability (longer beam traverse latencies and increased number of foot slips) with age and beam difficulty compared with control group of WT mice. The significant differences (*p *< 0.05) between beam performance of control YAC128 and control WT groups were observed at 9, 11 and 13 months of age on 11 mm round beam (Fig 2A, B); and at 6, 9, 11 and 13 months of age on 5 mm square beam (Fig 2C, D). Feeding TBZ to WT mice had no significant effects on beam performance of these mice (Fig 2A-D). However, feeding TBZ to YAC128 mice improved their beam-walking performance, significantly (*p *< 0.05) shortening the latencies and decreasing the foot slip numbers (Fig 2A-D). Significant differences (*p *< 0.05) of latency between YAC128 control group and early-TBZ-fed YAC128 group were detected at 9, 11, 13 months of age on 11 mm round beam (Fig 2A, B), and at 6, 9, 11 and 13 months of age on 5 mm square beam (Fig 2C, D). Late-TBZ feeding also significantly (*p *< 0.05) improved the beam performance of YAC128 mice at 9, 11 and 13 months of age on both 11 mm round beam (Fig 2A, B) and 5 mm square beam (Fig 2C, D). While conducting beam-walking assays, we noticed that some aging mice exhibited periods of "crawling behavior" (defined as prolonged contact between the thorax and abdomen of the mice and beam surface, with the mice using forelimbs to drag themselves along the beam). Three mice in YAC128 control group crawled on 11 mm round and 5 mm square beams at 11, 13 months time point. In contrast, none of the mice in WT groups or in YAC128 TBZ groups exhibited crawling behavior or fell off the beams at any age tested.

**Figure 3:**
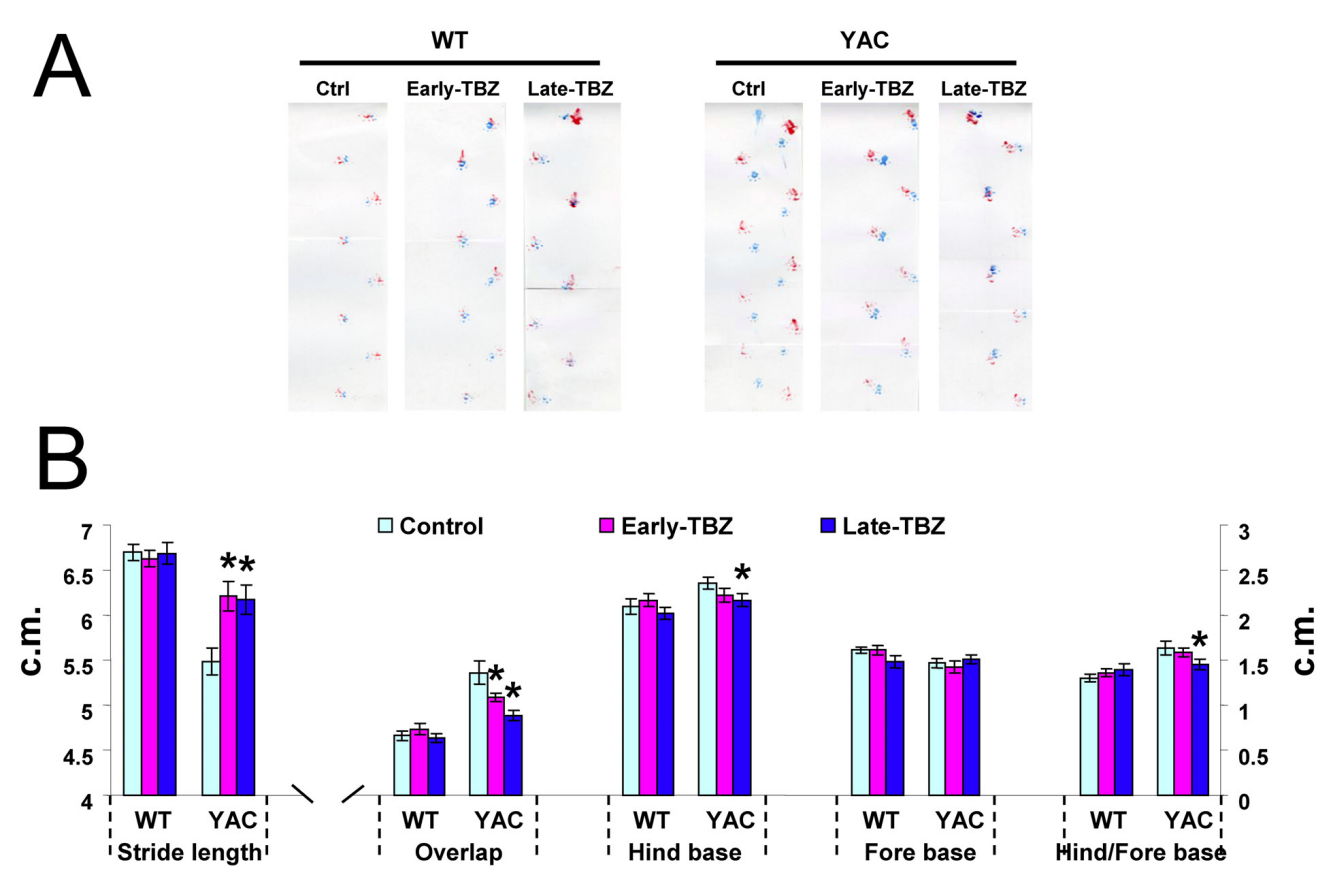
**Gait analysis of WT and YAC128 mice**. **A**, The footprint patterns of 13-month-old YAC128 (YAC) and WT mice. The footprints for control, Early-TBZ, and Late-TBZ groups are shown for both WT and YAC128 mice. **B**, The footprint patterns were assessed quantitatively by five measurements: stride length, hindbase width, frontbase width, front/hind footprint overlap and the ratio of Hindbase and Forebase. 13-month-old control YAC128 mice exhibited shorter stride lengths and increased front/hind paw overlap compared with control WT mice. Feeding TBZ to YAC mice improved their stride lengths and overlap. For the hindbase width, Late-feeding TBZ to YAC mice improved a little bit compared with control group. The frontbase width measurements are similar for all six groups of mice. The ratio of hindbase and forebase showed similar results with hindbase width analysis. Data for each measure are presented as mean ± SEM. * *p *< 0.05, significantly different from control YAC128 group.

At the conclusion of rotarod and beam-walking behavioral experiments (13 months of age), we also assessed gait abnormalities in all six groups of mice by footprint pattern analysis (Fig 3A). The footprint patterns were assessed quantitatively by five measurements: stride length, hindbase width, frontbase width, front/hind footprint overlap and the ratio of hindbase and forebase as we previously described [30,36]. We found that 13-month-old control YAC128 mice exhibited shorter stride lengths and increased front/hind paw overlap compared with control WT mice (Fig 3B). Feeding TBZ to YAC128 mice improved their stride lengths and overlap (Fig 3B). Our analysis further revealed that, for the hindbase width, Late-feeding TBZ to YAC mice slightly improved when compared with control YAC group (Fig 3B). The frontbase width measurements are similar in all six groups of mice. The ratio of hindbase and forebase showed similar results with hindbase width analysis (Fig 3B).

The combined results from "rotarod" (Fig 1), "beam-walk" (Fig 2), and "gait walk" (Fig 3) behavioral analysis lead us to conclude that both "early" and "late" TBZ feeding significantly alleviates motor deficits developed by aging YAC128 mice.

### Depression-related behaviors in TBZ-fed mice

**Figure 4:**
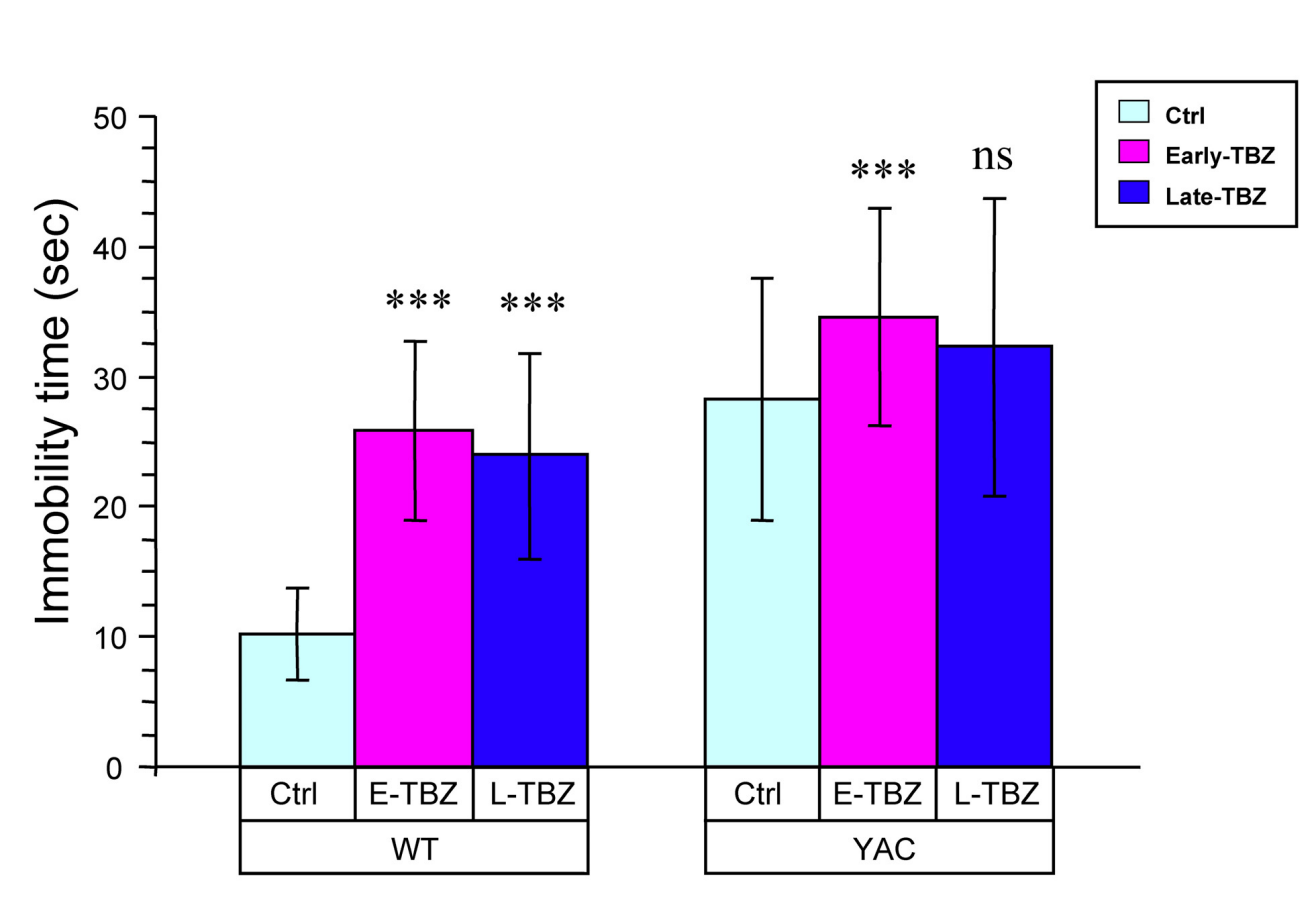
**Forced-swim test (FST) evaluation of WT and YAC128 mice**. 11-month-old mice were individually placed into a beaker of water (23-25°C) for a total of 300 secs and videotaped. Total immobility time of each mouse after the first 120 secs was manually scored by an experienced experimenter who was blinded to the genotype and drug treatment of the mice. YAC control group recorded significantly greater FST immobility time than WT control group (*p *< 0.05). Feeding TBZ to WT mice increased immobile than WT control mice (*p *< 0.05). Early-feeding TBZ to YAC mice exhibited more immobile compared with control YAC mice (*p *< 0.05). Late-feeding TBZ to YAC mice had no significant effect on depression-related behaviors compared with control YAC mice. ns, not significant.

In the process of behavioral testing we noticed that behavioral performance of Early-TBZ feeding groups (both WT and YAC) became impaired between 6, 9 and 11 months of age (Figs 1 and 2). We also noticed that the mice in Early-TBZ feeding groups (both WT and YAC128) appear to exhibit some symptoms of depression, such as hypoactivity and immobility in a tail suspension test. We started to observe these behavioral signs at ~7 months of age. We have not observed this phenomenon in the previous study [30]. In the current study we used 1.5-fold higher TBZ dosage compared to the previous study (based on TBZ blood concentration measurements). Also, mice in the current study were fed with TBZ three times per week compare with twice per week in the previous study. In addition, in the previous study we used TBZ + L-Dopa combination and now we used TBZ alone. Most likely all these factors contributed to "depression behavior" observed in the current study and not in the previous study. This "depression phenomenon" is of great interest since many HD patients taking TBZ become severely depressed [32,38]. Thus, we performed formal evaluation of these mice at 11 months of age using Forced-Swim Test (FST) depression behavior paradigm. We found that at 11 months of age YAC128 control group recorded significantly greater FST immobility time than WT control group (Fig 4). These results are consistent with previous description of "depression symptoms" in YAC128 mice using FST and additional behavioral paradigms [39]. We further found that feeding TBZ to WT mice significantly increased time of immobility in FST test (Fig 4). Early TBZ feeding to YAC128 mice resulted in increased time of immobility when compared to control YAC128 mice (Fig 4). Late TBZ feeding to YAC128 mice also increased time of immobity, but had less effect in FST assay than early TBZ feeding (Fig 4).

### TBZ protects against striatal cell loss in YAC128 HD mice

**Figure 5:**
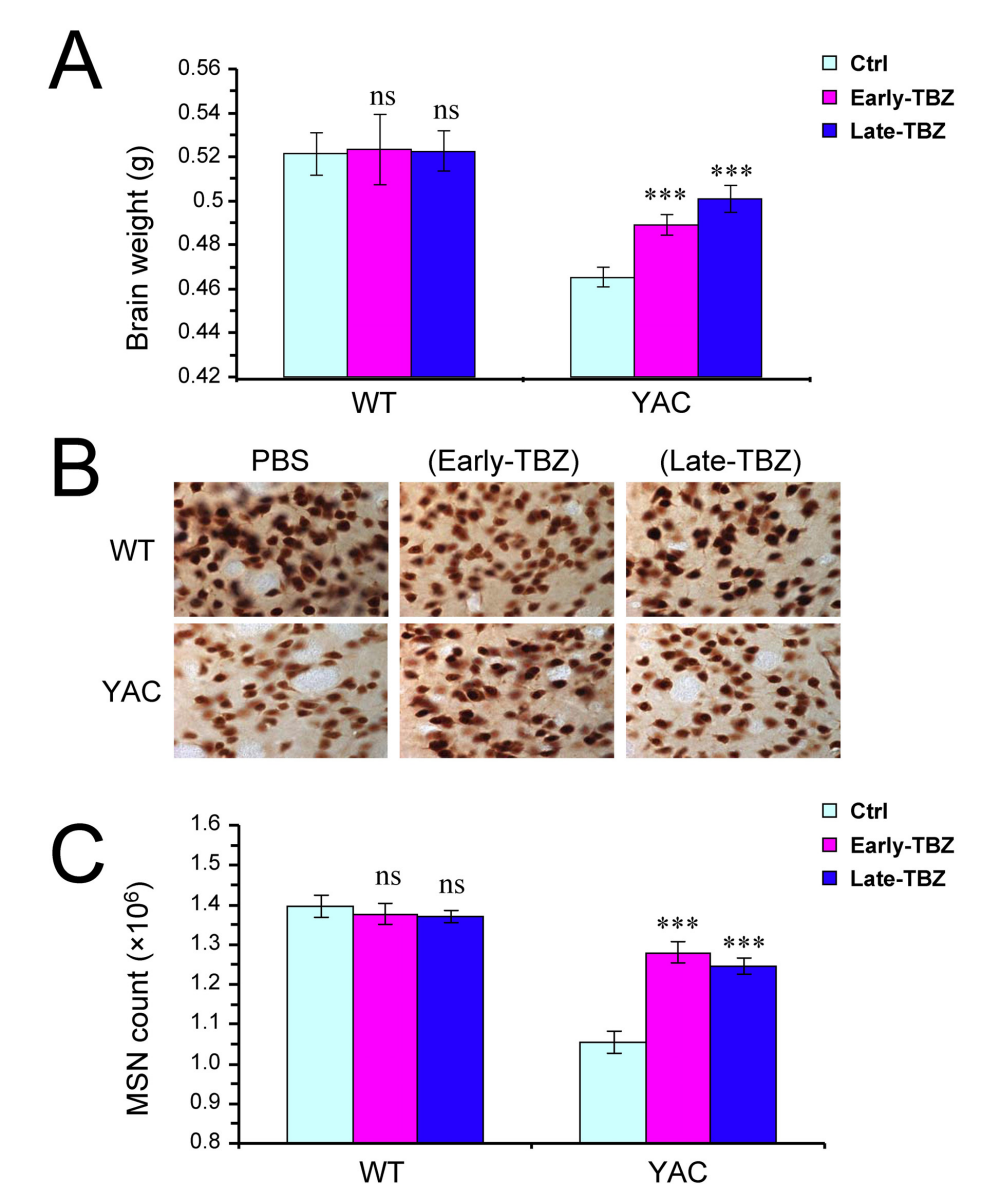
**Neuropathological analysis of WT and YAC128 mice**. **A**, Average brain weight of 13-month-old WT and YAC128 (YAC) mice. The brain weight of control YAC128 is significantly reduced compared with control WT group (*p *< 0.05). The brain weight of YAC128 mice fed with TBZ is significantly increased compared with brain weight of control (Ctrl) YAC128 mice (*** *p *< 0.05). **B**, Representative NeuN staining of striatal sections from 13-month-old WT and YAC128 mice. **C**, Average striatal neuronal counts of 13-month-old WT and YAC128 mice. Control YAC128 mice showed significant striatal neuronal loss (*p *< 0.05) compared with control WT mice. YAC128 mice fed with TBZ display significantly increased striatal neuronal counts compared with control YAC128 mice (*** *p *< 0.05) but significantly reduced neuronal counts compared with WT mice (*** *p *< 0.05). **A**, **C**, For each group of mice, the results are shown as mean ± SEM. Ctrl, Control.

To evaluate potential neuroprotective effects of TBZ, at the conclusion of behavioral trail (13 months time point) the brains from all 6 groups of experimental mice were removed from skull and weighted after transcardial perfusion. We found that the brains of control YAC128 mice weighted significantly less (*p *< 0.05) than the brains of control WT mice group (Fig 5A). Feeding TBZ to WT mice had no significant effect on brain weight of these mice (Fig 5A), whereas feeding TBZ to YAC128 mice resulted in significant increase (*p *< 0.05) in brain weight of these mice compared with control YAC128 (Fig 5A).

To obtain quantitative information about neuronal loss in these mice, the neuropathological assessments were performed by unbiased stereology as we previously described [30,36]. The brains were fixed, frozen, and sliced with the microtome, and coronal sections corresponding to striatal region were stained with monoclonal antibodies against NeuN protein. Representative NeuN staining of striatal sections from each group of 13-month-old WT and YAC128 mice are shown on Fig 5B. The number of NeuN-positive neurons in the striatum was counted blindly with respect to the nature of the slices (genotype and drug treatment). By stereological analysis, we determined that control YAC128 mice showed significant striatal neuronal loss (*p *< 0.05) compared with control WT mice (Fig 5C). We further found that feeding of TBZ to WT mice did not cause significant changes in striatal neuronal counts in these mice (Fig 5C). However, TBZ feeding significantly increased striatal neuronal counts (*p *< 0.05) in YAC128 mice (Fig 5C), indicating that TBZ protects YAC128 MSNs from cell death. Brain weight and MSN cell numbers were not significantly different between Early-TBZ and Late-TBZ groups of YAC128 mice, with both groups showing similar degree of improvement when compared to control YAC128 group (Fig 5A, C). The results of our neuropathological analysis indicated that "early" and "late" feeding with TBZ significantly protected striatal neurons from cell death in aging YAC128 mice.

## Discussion

Striatum is the predominant target of midbrain dopaminergic neurons [12], and a number of experimental evidence point to a connection between dopamine signaling and MSN neurodegeneration in HD [26]. Striatal MSN neurons expressing dopamine receptors in the striatum predominantly degenerate in HD patients, with cerebral cortex neurons being affected to a much lower extent, and striatal interneurons being spared [40,41]. Biochemical analysis revealed progressive loss of striatal D1 and D2 receptors in post mortem HD brains [42-46]. Imaging studies also reported reduction of striatal D1 and D2 receptors in HD patients [13,14] and in asymptomatic HD mutation carriers [15-19,47,48]. Consistent with analysis of human HD cases, drastic reduction of striatal D1 and D2 receptor density [19,20] and deficiencies in D1 receptor-mediated signaling [21] were observed in R6/2 HD mouse model prior to onset of degeneration. In cellular models dopamine potentiates mutant huntingtin-mediated neurotoxicity by acting via D1-class [30,31] and D2-class [25] dopamine receptors. The HD-like motor dysfunction and selective MSN degeneration have been observed in the dopamine transporter knockout mice [24]. The hyperdopaminergic transmission accelerated formation of Htt^exp ^aggregates in 92Q knock-in HD mouse model [29]. In our previous study we reported that feeding L-Dopa to YAC128 mice accelerated progression of the motor phenotype and increased neuronal loss in these mice [30]. Most of these findings are consistent with an idea that dopamine exerts toxic effects on striatal neurons in the context of HD mutation, which leads in compensatory loss of D1 and D2 receptors in striatal region of HD brains.

Striatal MSN loss is the hallmark of HD. Increasing evidence suggested a permissive role of dopaminergic innervation of the striatum in the excitotoxicity in HD. Elevated glutamate-induced Ca^2+ ^signals had been found to play an important role in HD [9], and dopamine could significantly potentiate glutamate-induced Ca^2+ ^signals and MSN death in HD model [30], suggesting that glutamate and dopamine signaling pathways act synergistically to induce elevated Ca^2+ ^signals and to cause apoptosis of HD MSNs [30]. D1 class DARs are coupled to Ca^2+ ^signalling via Gs/olf- cAMP-PKA, PKA activation modulates the glutamate-related Ca^2+ ^signaling pathway by facilitating the activity of NMDAR [49,50], AMPAR [51], and InsP_3_R1 [52,53]. D2 class DARs directly coupled to Ca^2+ ^signalling in MSNs via PLC activation. Therefore both D1 and D2 DARs might be involved in the potentiation effects of dopamine on elevated Ca^2+ ^signalling and cell death in HD MSNs, and blockade of both D1 and D2 DARs would be necessary to exert significantly clinical beneficial effects for dopamine antagonist treatment in HD.

Ca^2+ ^signalling is an important downstream in dopamine signalling cascades. In HD MSN, mutant Htt^exp ^potentiates the activity of NR2B NMDAR [8,54-57] and InsP_3_R1 [58], which in turn causes abnormal function and cell death of HD MSN due to the disturbed Ca^2+ ^signals. Thus over activation of Ca^2+ ^signaling by mutant Htt^exp ^may lead to the compensatory loss of D1 and D2 receptors in striatal region of HD brains. Indeed, a significant reduction of striatal D1 and D2 receptors was found in PET imaging studies [13,14] in HD patients and in HD mouse models [19-21]. The idea of the compensatory loss of D1 and D2 receptors are further supported by our rencent behavioral evaluation of dopamine tone in aging YAC128 HD mice (Wu at al, in preparation). Decreased denisty of striatal monoaminergic terminals was found in HD patients using DTBZ ((+)-alpha- [11C]dihydrotetrabenazine) PET imaging technique [59], suggesting the deficits of presynaptic dopamine innervation in HD. However, unlike the significant presynaptic nigrostriatal dopaminergic denervation in Parkinson's disease, Huntington's disease is characterized by more prominent striatal dopamine receptor loss, whereas nigrostriatal denervation is present to a lesser degree [60]. We explain the decrease of dopaminergic inputs in HD as a compensatory effect of dopamine receptor loss in late stage of HD.

Tetrabenazine (TBZ) is a potent blocker of vesicular monoamine transporter (VMAT2). TBZ causes depletion of dopamine content in the presynaptic vesicles and reduction in the dopaminergic tone. In previous clinical trials TBZ has been shown to significantly reduce chorea symptoms in HD patients when compared with placebo group [32-35]. In our previous experiments with YAC128 mouse model we demonstarted that long term administration of TBZ (combined with L-Dopa) alleviated motor deficits and reduced striatal cell loss in these mice [30]. These results indicated that TBZ and possibly other dopamine signaling antagonists may have a therapeutic potential for treatment of HD beyond previously established "symptomatic" benefit. In 2008 TBZ became the first drug officially approved by the Food and Drug administration for treatment of HD patients in the United States [35], greatly enhancing the need to carefully characterize actions of this drug in the context of HD models. We now repeated evaluation of TBZ in YAC128 model of HD and compared "early" (2 months) and "late" (6 months) treatment groups. We found that both "early" and "late" TBZ treatments alleviated the motor deficits (Figs 1, 2 and 3) and reduced striatal cell loss (Fig 5) in YAC128 mice, suggesting that treatment of both presymptomatic and early symptomatic HD patients with TBZ may have neuroprotective effects and delay progression of the disease. We should mentioned here that, TBZ is not a specific dopamine depleting agent, other neurotransmitter innervations such as serotoninergic and some noradrenergic may also play a role in this neuroprotective effects of TBZ.

By blocking VMAT2 TBZ depletes biogenic amines, including dopamine as well as serotonin and norepinephrine. Reduced levels of serotonin can cause depression. Indeed, it has been reported that many HD patients taking TBZ became severely depressed [32,38]. Interestingly, we noticed that TBZ treatment groups of WT and YAC128 mice exhibited some symptoms of depression, such as hypoactivity and immobility in a tail suspension test. We performed a formal depression behavior analysis at 11 months of age for all 6 groups of mice by using forced-swim test (FST). In this analysis we discovered that both "early" and "late" TBZ treatment groups of WT and YAC128 mice appear to be depressed when compared to control groups (Fig 4). Thus, we concluded that TBZ is a useful therapeutic for treatment of HD, however prolonged treatment with TBZ induces depression. Additional dopamine antagonists which do not interfer with serotonin signaling system should be evaluated as potential HD therapeutics. The paradigms described in the present study can be used for evaluation of beneficial effects of these compounds for HD treatment as well as their ability to cause depression-like behaviors. A novel compound huntexil (pridopidine; ACR16) is a modulator of D2 receptor activity [61,62] which has been recently developed by NeuroSearch for treatment of movement and psychiatric disorders. In recently completed phase III HD clinical trial (MermaiHD study), Huntexil demonstarted significant clinical benefit http://www.neurosearch.com/Default.aspx?ID=16&M=News&PID=12&NewsID=15886. Importantly, further analyisis of results of phase III trial suggested that Huntexil exerted not only symptomatic benefit but was also able to slow the underlying disease progression http://www.neurosearch.com/Default.aspx?ID=16&M=News&PID=12&NewsID=15894. These clinical findings with Huntexil are consistent with disease-modifying effects of TBZ that we observed in the present and previous studies with YAC128 HD mouse model (Fig 5 and [30]). It will be of interest to compare huntexil with TBZ and with other clinically relevant D1 and D2 receptor antagonists in YAC128 mouse model by following procedures described in the present report. Obtained results will provide opportunity to systematically compare symptomatic and disease modifying effects of these dopamine antagonists in HD, as well as evaluate potential side effects such as induction of depression.

## Conclusions

Our present study demonstrated that TBZ, a dopamine signaling antagonist have therapeutic potential for treatment of HD beyond previously established "symptomatic" benefit. We found that both "early" and "late" TBZ treatments alleviated the motor deficits and reduced striatal cell loss in YAC128 mice, suggesting that treatment of both presymptomatic and early symptomatic HD patients with TBZ may have neuroprotective effects and delay progression of the disease. Moreover, we have been able to recapitulate and quantify depression-like symptoms in TBZ-treated mice, reminiscent of common side effects observed in HD patients taking TBZ, highlighting the need to develop and evaluate more specific dopamine antagonists for HD treatment.

## Methods

### Drug delivery in mice

All animal studies were approved by the University of Texas Southwestern Medical Center Animal Care and Use Committee. YAC128 mice (FVBN/NJ background strain) [37] were obtained from Jackson Labs (stock number 004938). Age-matched female wild type and YAC128 hemizygotous littermate mice were used in all our experiments. Tetrabenazine (TBZ) was obtained from Tocris, mixed with 2% cornflour using ceramic grinder and resuspended in PBS. TBZ was delivered to mice by oral feeding approach that we used in our previous studies with YAC128 mice [30]. The drugs were fed orally to mice three times a week starting at 2 months of age. The mice were fed with 0.125 mg of TBZ suspended in 50 μl of PBS with 2% corn flour. To determine the efficacy of this drug delivery procedure, 7 wild-type mice of 2 months of age were fed orally with 0.125 mg of TBZ formulation and the blood samples were collected 30 minutes after drug feeding. The samples were diluted 1:1 in water for hemolyzation, flash frozen and shipped to Melior Discovery (Exton, PA) for quantitative analysis. The TBZ levels were analyzed using HPLC and compared with a standard TBZ sample. Data are expressed as average of the values from five samples ± SEM (see Results).

### Motor coordination assessments in mice

The motor coordination experiments were performed as previously described [30,36] with minor modifications. The "beam-walking" assay was performed using a home-built experimental setup. The 17 mm round beam, 11 mm round beam, and 5 mm square beam were used for training. At each time point (2, 6, 9, 11 and 13 months of age), the mice were trained to traverse the beam to the enclosed box. The mice were trained on 17 mm round beam for the 1st day, 11 mm round beam for the 2nd day, and 5 mm square beams for the 3rd day (two trials per day). Once the stable baseline of performance was obtained, the mice were tested in two consecutive trials on 11 mm round beam and then on 5 mm square beam, in each case progressing from the widest to the narrowest beam. The latency to traverse the middle section (80 cm in length) of each beam and the number of times the hind feet slipped off each beam were recorded for each trial. For each measurement, the mean scores of the two trials for each beam were used in the analysis.

The rotarod assessments were performed using Economex rotarod apparatus (Columbus Instruments, Columbus, OH) as previously described [30]. At each time point, the mice were trained on the accelerating rotarod (accelerated from 0 to 40 rpm over 200 s) with 2 trials per day for 3 consecutive days, by which time a steady baseline level of performance was attained. The testing was executed over 1 day with 1.5 h of rest between tests. The mean latency to fall off the rotarod recorded in the two trials is used in analysis.

For the footprint test, the forepaws and hindpaws of the mice were coated with red and green nontoxic paints, respectively. The mice were trained to walk along a 50-cm-long, 10-cm-wide, paper-covered runway (with 10-cm-high walls) into an enclosed box. All the mice were given two runs per day for 3 consecutive days. A fresh sheet of white paper was placed on the floor of the runway for each run. The footprint patterns were assessed quantitatively by five measurements: stride length, hindbase width, frontbase width, front/hind footprint overlap and the ratio of hindbase and forebase as we previously described [30].

### Forced swim test

Immobility in the forced swim test (FST) is a commonly used for measurement of depression in rodents [63-65]. Mice were individually placed into an individual glass beaker (54 cm in height and 24 cm in diameter) filled with room temperature water (23-25°C) to 40 cm depth. All test sessions were recorded for a total of 300 seconds by a video camera from the side of the cylinder. Total immobility time of each mouse after the first 120 seconds was manually scored by an experienced experimenter who was blinded to the genotype and drug treatment of the mice. Immobility was defined as the state in which mice were judged to be making only the movements necessary to keep their head above the surface.

### Neuropathological assessments in mice

The neuropathological assessments were performed as previously described [30]. At conclusion of behavioral testing (13 months time point), the mice were terminally anesthetized and perfused transcardially with 10 ml of 0.9% saline followed by 100 ml of fixative (4% paraformaldehyde in 0.1 M PBS, pH 7.4). All brains were removed from the skull, weighed, and transferred to postfixative overnight at 4°C in 4% paraformaldehyde and equilibrated in 20-30% (w/v) sucrose in PBS. The brains were processed and cut into 30-μm-thick coronal sections as described above. The coronal sections spaced 360 μm apart throughout the striatum (in the range from +1.70 mm to -2.30 mm relative to bregma) were stained with NeuN monoclonal antibody (1:1000 dilution; Millipore, Billerica, MA) and biotinylated anti-mouse secondary antibodies (1:200 dilution; Vector Laboratories, Burlington, Ontario, Canada) (M.O.M kit). Signal was amplified with an ABC Elite kit (Vector Laboratories) and detected with diaminobenzidine (Vector Laboratories). All quantitative stereological analyses were performed blindly with respect to the nature of slices (genotype and drug feeding) using Stereoinvestigator setup and software (MicroBrightField, Williston, VT). The grid size was set to 450 × 450 μm, and the counting frame was 50 × 50 μm. The average slice thickness after histological processing was determined to be 25 μm.

### Statistical data analysis

The data were analyzed using SAS 9.13. The rotarod performance and beam-working performance were analyzed using 3 way ANOVA accounting for gene type (YAC128 vs wild type), drug treatment (PBS vs Early or Late-TBZ feeding) and animals age for all factor analysis, 2 way ANOVA for comparing treatment effects of Early or Late-TBZ feeding vs PBS control, or Early or Late-TBZ feeding in YAC128 and WT respectively, and 1 way ANOVA for age effects in animals treated with PBS alone. Tukey test or Student t test were also used where applicable.

## Competing interests

The authors declare that they have no competing interests.

## Authors' contributions

HW designed the study, participated in the experiments, performed the statistical analysis and wrote the manuscript. XC and YL participated in the behavioral study and neuropathological analysis. IB and TST conceived of the study, participated in its design and coordination and helped to draft the manuscript. All authors read and approved the final manuscript.
